# Wild-type SARS-CoV-2 neutralizing immunity decreases across variants and over time but correlates well with diagnostic testing

**DOI:** 10.3389/fimmu.2023.1055429

**Published:** 2023-02-08

**Authors:** Kelly M. O’Shea, Charles F. Schuler, Jesse Chen, Jonathan P. Troost, Pamela T. Wong, Kelsea Chen, Daniel R. O’Shea, Westley Peng, Carmen Gherasim, David M. Manthei, Riccardo Valdez, James L. Baldwin, James R. Baker

**Affiliations:** ^1^Division of Allergy and Clinical Immunology, Department of Internal Medicine, University of Michigan, Ann Arbor, MI, United States; ^2^Mary H. Weiser Food Allergy Center, University of Michigan, Ann Arbor, MI, United States; ^3^Michigan Nanotechnology Institute for Medicine and Biological Sciences, University of Michigan, Ann Arbor, MI, United States; ^4^Michigan Institute for Clinical and Health Research, University of Michigan, Ann Arbor, MI, United States; ^5^Department of Pathology, University of Michigan, Ann Arbor, MI, United States

**Keywords:** COVID-19, SARS-CoV-2, antibody, viral neutralization, variant of concern, spike, nucleocapsid, vaccine

## Abstract

**Importance:**

The degree of immune protection against severe acute respiratory syndrome coronavirus-2 (SARS-CoV-2) variants provided by infection versus vaccination with wild-type virus remains unresolved, which could influence future vaccine strategies. The gold-standard for assessing immune protection is viral neutralization; however, few studies involve a large-scale analysis of viral neutralization against the Omicron variant by sera from individuals infected with wild-type virus.

**Objectives:**

1) To define the degree to which infection versus vaccination with wild-type SARS-CoV-2 induced neutralizing antibodies against Delta and Omicron variants.

2) To determine whether clinically available data, such as infection/vaccination timing or antibody status, can predict variant neutralization.

**Methods:**

We examined a longitudinal cohort of 653 subjects with sera collected three times at 3-to-6-month intervals from April 2020 to June 2021. Individuals were categorized according to SARS-CoV-2 infection and vaccination status. Spike and nucleocapsid antibodies were detected *via* ADVIA Centaur^®^ (Siemens) and Elecsys^®^ (Roche) assays, respectively. The Healgen Scientific^®^ lateral flow assay was used to detect IgG and IgM spike antibody responses. Pseudoviral neutralization assays were performed on all samples using human ACE2 receptor-expressing HEK-293T cells infected with SARS-CoV-2 spike protein pseudotyped lentiviral particles for wild-type (WT), B.1.617.2 (Delta), and B.1.1.529 (Omicron) variants.

**Results:**

Vaccination after infection led to the highest neutralization titers at all timepoints for all variants. Neutralization was also more durable in the setting of prior infection versus vaccination alone. Spike antibody clinical testing effectively predicted neutralization for wild-type and Delta. However, nucleocapsid antibody presence was the best independent predictor of Omicron neutralization. Neutralization of Omicron was lower than neutralization of either wild-type or Delta virus across all groups and timepoints, with significant activity only present in patients that were first infected and later immunized.

**Conclusions:**

Participants having both infection and vaccination with wild-type virus had the highest neutralizing antibody levels against all variants and had persistence of activity. Neutralization of WT and Delta virus correlated with spike antibody levels against wild-type and Delta variants, but Omicron neutralization was better correlated with evidence of prior infection. These data help explain why ‘breakthrough’ Omicron infections occurred in previously vaccinated individuals and suggest better protection is observed in those with both vaccination and previous infection. This study also supports the concept of future SARS-CoV-2 Omicron-specific vaccine boosters.

## Introduction

Severe acute respiratory syndrome coronavirus-2 (SARS-CoV-2), the causative virus for the coronavirus disease 2019 (COVID-19) pandemic, has caused millions of cases and deaths worldwide (1). Throughout the pandemic, mutations of the original virus have led to new viral variants (2, 3), and these “variants of concern” have raised questions about pathogenesis and immune escape, especially as additional waves of infection have occurred.

Vaccination against COVID-19 is effective in mitigating severe disease and hospitalization from SARS-CoV-2 (4–7). Furthermore, infection with SARS-CoV-2 induces strong cellular and humoral immunity, with the magnitude of response possibly correlating with disease severity (8–10). However, reinfections have raised questions pertaining to protective immunity after infection and vaccination, especially with Omicron variants (11–14). Current primary vaccines for SARS-CoV-2 target the wild-type (WT) spike protein, and studies have documented reduced viral neutralization titers to Omicron as compared to other variants (13). However, studies are necessary to clarify how the combination of WT infection and vaccination impact viral immune responses to subsequent viral variants in order to define the value of including additional spike protein SARS-CoV-2 variants in vaccine boosters.

Viral neutralization assays evaluate the functional ability of antibodies to neutralize SARS-CoV-2 and provide insights into immune protection against infection (15). Unfortunately, these assays are cumbersome because they must be performed in biosafety level 3 facilities, requiring intensive time, resources, and expertise. Pseudoviral neutralization of lentiviruses expressing SARS-CoV-2 spike protein is an effective surrogate to standard neutralization assays (16, 17), however even this technique is too complex for clinical deployment. Therefore, most clinical laboratory tests for SARS-CoV-2 humoral immunity involve simply identifying and/or quantifying overall spike and nucleocapsid antibody levels rather than their neutralizing functional capacity.

To better understand the infection and vaccination factors leading to protection from SARS-CoV-2, we conducted a large population study to assess the neutralizing capacity of sera from individuals with varied WT COVID-19 disease and vaccination status. We also defined the ability of these individuals to neutralizing subsequent variants, including Omicron. Further, we attempted to determine whether clinically available serological assays can provide insight into variant neutralization capability.

## Methods

### Study populations

**Figure 1:**
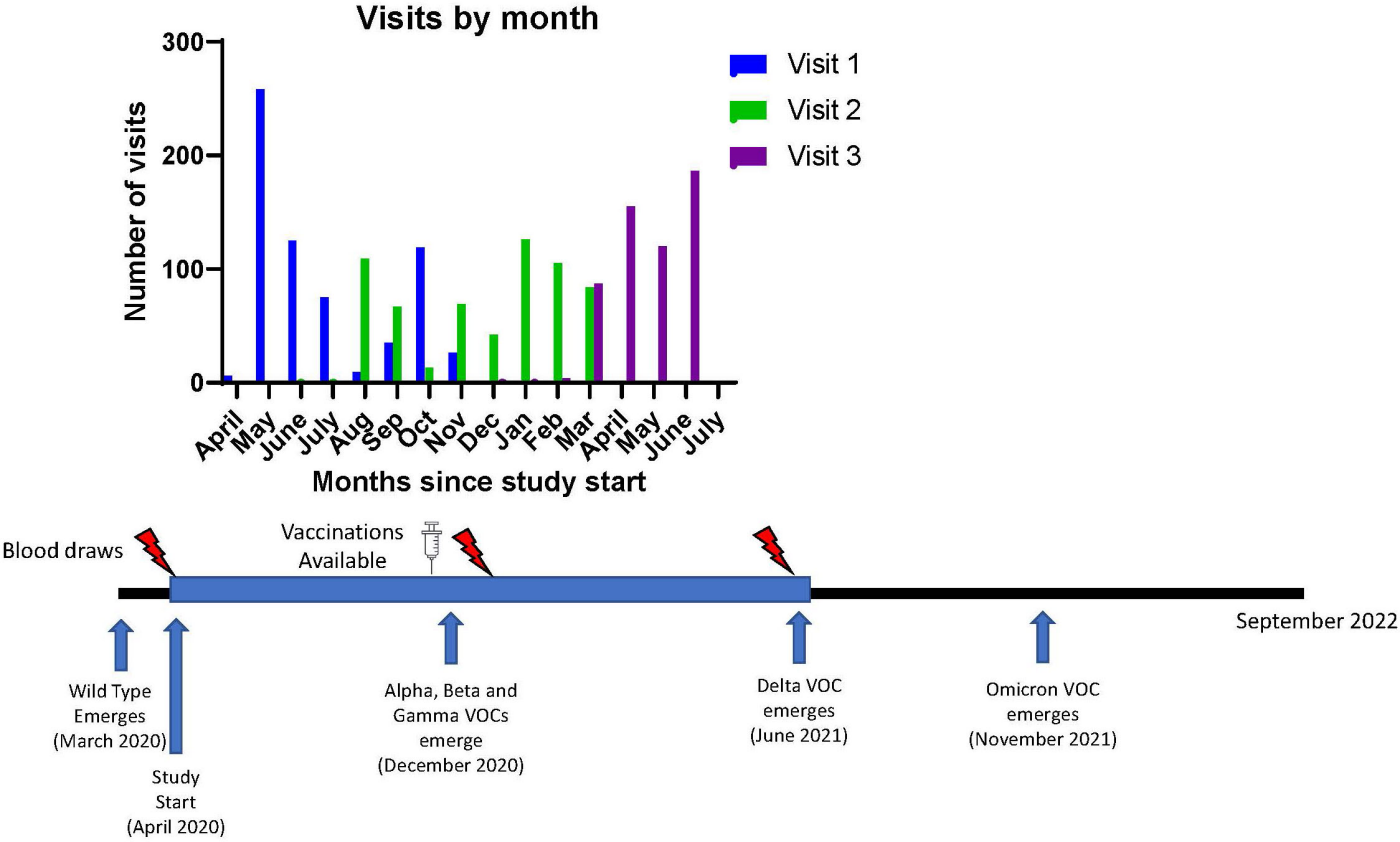
Study Timeline and Emergence of known Variants of Concern for SARS-CoV-2. Timeline of visits related to vaccine availability and variant detection dates. VOC, variant of concern.

The University of Michigan (U-M) Institutional Review Board approved this study (HUM00180074). All subjects provided written informed consent. Additional details on recruitment, inclusion/exclusion criteria, data collection, and specimen processing are available from prior reports on this cohort (10, 18). Subjects underwent 3 visits, each approximately 3-6 months apart and serum was collected at each visit (**Figure 1**). All study visits were concluded by 6/24/2021 while the Delta variant became a variant of concern in the United States on 6/15/2021 (19).

### Vaccination

During the study, both COVID-19 mRNA vaccines [BNT162b2 (Pfizer) or mRNA-1273 (Moderna)] became available. Participants who chose to be vaccinated remained in the study. The vaccination type and administration dates were recorded. Booster third vaccine doses were not available during the study timeframe.

### Subject categorization

Subjects were categorized into 4 groups according to SARS-CoV-2 and vaccination status; SARS-CoV-2 infection positive/vaccine positive (I+/V+), SARS-CoV-2 infection positive/vaccine negative (I+/V-), SARS-CoV-2 infection negative/vaccine positive (I-/V+), and SARS-CoV-2 infection negative/vaccine negative (I-/V-). Vaccination status was considered positive for individuals that had completed the 2-dose series of a COVID-19 mRNA vaccination. Infection status was considered positive if an individual had a reverse transcriptase polymerase chain reaction (RT-PCR) positive for SARS-CoV-2 from a nasopharyngeal swab or evidence of a positive nucleocapsid (N) antibody and was considered negative if an individual had no history of a positive RT-PCR and negative nucleocapsid antibody testing. Individuals were categorized according to infection and vaccination status independently at each timepoint. For all participants in this cohort, infection occurred prior to vaccination.

### SARS-CoV-2 nucleocapsid electrochemiluminescence immunoassay and spike (S1-RBD) chemiluminescence immunoassay

The Elecsys^®^ (Roche) SARS-CoV-2 Total Antibody Assay on a Cobas e411 analyzer was used to detect anti-nucleocapsid antibodies. The ADVIA Centaur^®^ (Siemens) SARS-CoV-2 Total (COV2T) assay on an ADVIA Centaur^®^ XPT analyzer was used to detect anti-spike (S) antibodies. These assays detect total SARS-CoV-2 nucleocapsid or S antibodies *via* a sandwich electrochemiluminescence immunoassay or a chemiluminescence immunoassay, respectively. A cutoff index of >1 is defined as a positive result. The assays detect all isotypes in aggregate of the relevant SARS-CoV-2 antibody. All samples were evaluated in the CLIA-certified U-M Clinical Pathology Laboratory.

### SARS-CoV-2 lateral flow assay

A SARS-CoV-2 lateral flow immunoassay (LFA) from Healgen Scientific (COVID-19 IgG/IgM Rapid Test Cassette) was also used to separately evaluate IgG and IgM antibodies toward the SARS-CoV-2 S1-receptor binding domain. The assay was run following the Emergency Use Authorization - Instructions for Use (20) using subject serum. A faint line was read as positive. “IgG” refers to a positive IgG result and “IgM” refers to a positive IgM result from this LFA hereafter.

### Pseudoviral neutralization assay

SARS-CoV-2 wild-type (WT), B.1.617.2 (Delta) and B.1.1.529 (Omicron BA.1) spike proteins were cloned into a lentivirus vector as previously described (21). To perform pseudoviral neutralization assays, HEK-293T cells stably expressing human ACE2 were seeded at 9*10^3 cells per well of white clear-bottom, tissue culture 96-well plates and incubated at 37°C and 5% CO_2_ for 24 hours in complete DMEM (DMEM/10% FBS/1% Pen-Strep). Serum samples were serially diluted 2-fold in complete DMEM starting at an initial dilution of 1:10. Equal volumes of diluted sera were mixed with each pseudovirus expressing variant spike proteins diluted in DMEM with polybrene in a new 96-well plate, giving a final concentration of 4,163 transduction units/mL lentivirus with polybrene (8 ug/mL) with a serum starting dilution of 1:20. Sera and virus were incubated at 37°C and 5% CO_2_ for 1 hour. Complete DMEM in the HEK-293T plate was replaced with 100 uL of the serum-pseudovirus mixtures and then incubated at 37°C and 5% CO_2_ for 4 hours. Serum-pseudovirus mixtures were aspirated from the cell culture plates and replaced with 100 uL of fresh complete DMEM and then incubated at 37°C and 5% CO_2_ for 72 hours. Uninfected cell and virus only controls (without sera) were plated for all variants during each assay to standardize results. After 72 hours, luciferase detection was performed using a GloMax (Promega) plate reader according to manufacturer instructions. BrightGlo assay reagent (Promega, Madison, WI) was used for WT and Omicron pseudovirus neutralization and SteadyGlo (Promega) assay reagent was used for Delta variant pseudovirus neutralization to account for differences in luminescence with the different viral constructs.

### Statistical analysis

Descriptive statistics were provided using mean and standard deviation for continuous variables and frequencies and percentages for categorical variables. Fifty percent inhibitory concentration (IC50) analyses were performed in Prism V8.0 (GraphPad Inc., San Diego, CA). The datasets were not normally distributed based on histogram visualization and the Shapiro-Wilk test for normality. Statistical comparison between multiple groups was performed using Kruskal-Wallis Multiple Comparisons. Antibody variables of interest included IgM (positive/negative), IgG (positive/negative), Spike (positive/negative), Spike (continuous—with upper and lower limit values recoded as follow: <0.05 = 0.05 and >10.0 = 10.0), Nucleocapsid (positive/negative), Nucleocapsid (continuous). Linear mixed effects models of log-transformed IC50 values were developed with a random intercept for each subject and repeated measures for time with an auto-regressive covariance structure to address the repeated measures within subjects. Beta values reported reflect positive (>0) or negative (<0) associations between the dependent and independent variables, with no effect at beta = 0. Analyses and figure production were performed in SAS V9.4 (SAS Institute Inc., Cary, NC) and Prism V8.0 (GraphPad Inc., San Diego, CA).

## Results

### Patient characteristics

Descriptive statistics for the full study cohort are detailed in **Table 1**. The mean age was 40.6 ± 12.1 years. Female participants composed 72% of the cohort and healthcare workers composed 80%. 151 participants (23%) had pre-existing medical conditions. The most prevalent pre-existing conditions were chronic lung disease (10%) and hypertension (10%).

**Table 1:** Participant descriptive statistics.

Age (years)	
Mean (SD)	40.6 (12.14)
N	653
Sex, n (%)	
Female	472 (72)
Male	176 (28)
Other	3 (1)
Unknown/Not Reported	2 (0)
Race, n (%)	
Unknown/Not Reported	3 (1)
American Indian/Alaska Native	1 (0)
Asian	57 (9)
Black or African American	26 (4)
More Than One Race	18 (3)
Native Hawaiian or Other Pacific Islander	1 (0)
White	545 (84)
Ethnicity, n (%)	
Unknown/Not Reported	3 (1)
Hispanic or Latino	32 (5)
NOT Hispanic or Latino	618 (94)
BMI (kg/m)	
Mean (SD)	27.7 (8.33)
Enrollment Group, n (%)	
Healthcare worker	522 (80)
Not HC worker	131 (20)
Pre-existing Medical Conditions, n (%)	151 (23)
Chronic Lung Disease (asthma/emphysema/COPD)	66 (10)
Diabetes Mellitus	22 (3)
Cardiovascular Disease	13 (2)
Chronic Renal Disease	3 (<1)
Liver Disease	1 (<1)
Hypertension	66 (10)
Immunocompromised condition	4 (1)
Neurologic/Neurodevelopmental/Intellectual Disability	1 (<1)

### Viral neutralization is highest in individuals with both infection and vaccination history across all variants

**Figure 2:**
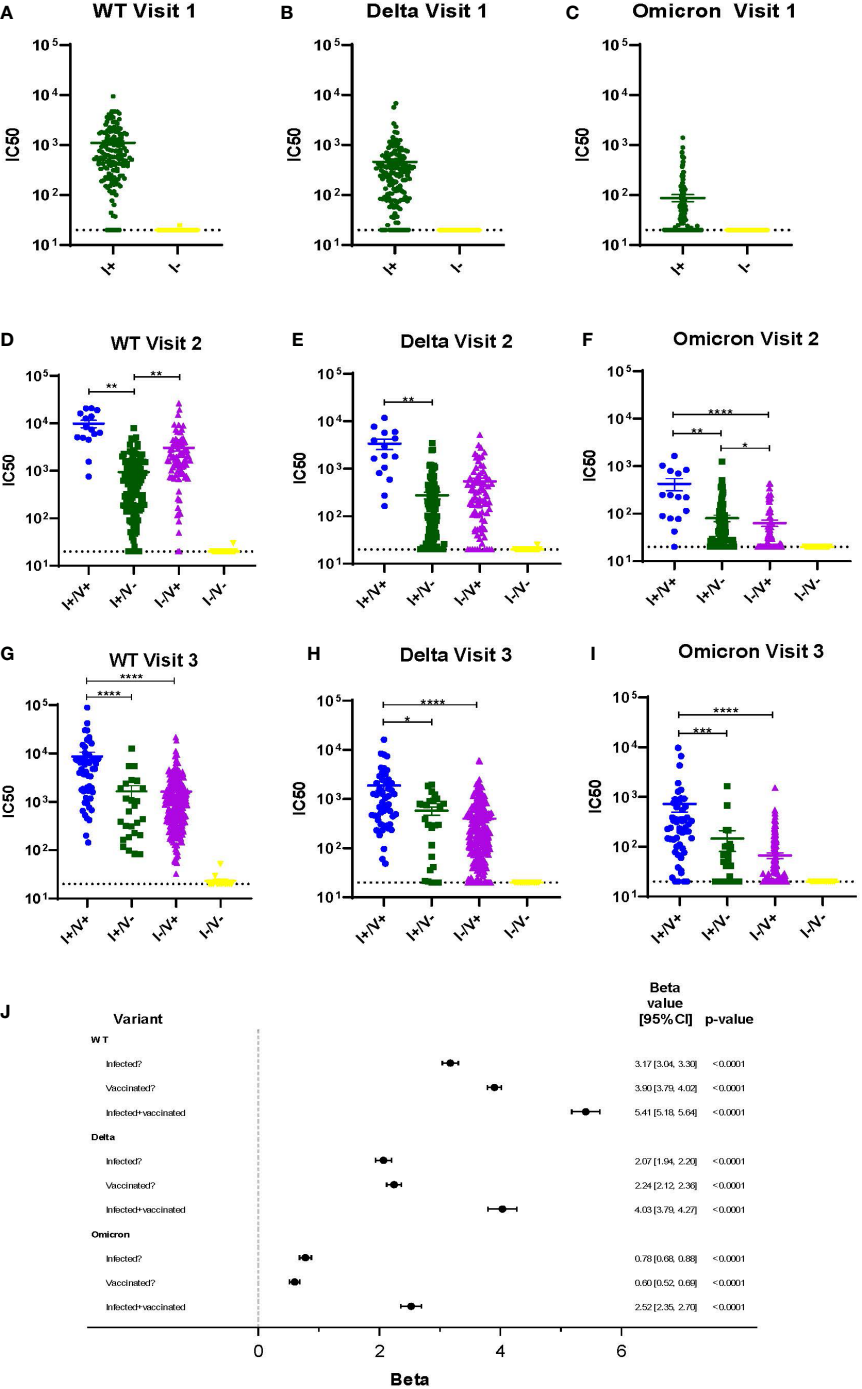
Viral neutralization across 3 timepoints for WT, Delta and Omicron SARS-CoV-2. **(A-I)** Viral neutralization across 3 timepoints for WT, Delta, and Omicron SARS-CoV-2. Each point represents the IC50 (log10 transformed) of an individual. Serum dilutions start at 1:20, visualized by the dotted line. Statistical analysis was performed using Kruskal-Wallis Multiple Comparisons. I, Infection; V, Vaccine; IC50, half maximal inhibitory concentration. **(J)**. Results of a linear mixed effects model of log-transformed IC50 values analyzed according to infection, vaccination, or infection plus vaccination status. *p ≤ 0.05, **p ≤ 0.01, ***p ≤ 0.001, ****p ≤ 0.0001.

Viral neutralization was performed on all serum samples at each timepoint using three SARS-CoV-2 variants (WT, Delta, and Omicron) and neutralization titers were compared across all variants and groups (**Figures 2A-I**). COVID-19 vaccination became available just prior to Visit 2. At Visits 2 and 3 for every variant, the average IC50 is highest amongst individuals with a history of COVID infection and who also had completed the 2-dose vaccination series (I+/V+) (**Figures 2D-I**). Individuals who had vaccination or infection alone had lower levels of neutralization. Not surprisingly, individuals who had no history of COVID infection and did not receive any vaccine doses (I-/V-) had the lowest average IC50 (**Figures 2A-I**). The average IC50 of all groups with either vaccination, infection or both (I+/V+, I+/V- and I-/V+) were significantly higher than the I-/V- group at every timepoint. By Visit 3, the I+/V+ group showed significantly greater neutralization activity than either the I+/V- or I-/V+ groups for all variants. There was no significant difference between the average IC50 for the I+/V- and I-/V+ groups at visit 3 for all variants.

A multivariable linear mixed effects model of log-transformed IC50 values was used to better assess the independent impacts of infection and vaccination status on individual serum ability to neutralize variant virus (**Figure 2J**). Across all variants, a history of infection, a history of vaccination, or both were all independent predictors of viral neutralization. However, the magnitude of effect was greatest in the infected and vaccinated group (I+/V+), although the impact decreased across later variants. For example, viral neutralization activity associated with the combination of vaccination and infection was only 74% of WT serum neutralization activity for Delta and was only 47% for Omicron.

**Figure 3:**
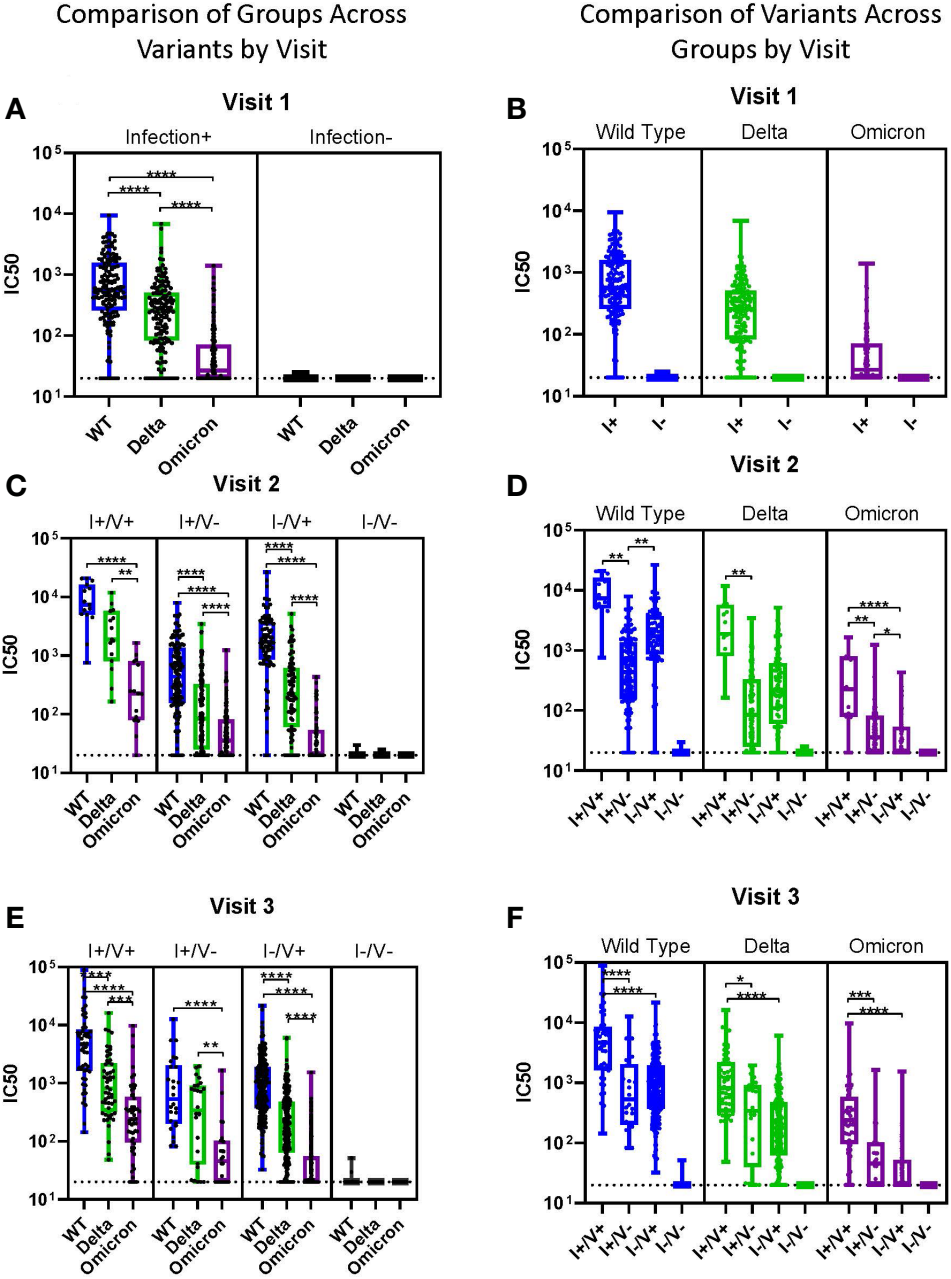
Impact of Variants by Group. **(A-F)** Viral neutralization comparisons of WT, Delta, and Omicron SARS-CoV-2. Each point represents the IC50 (log10 transformed) of an individual. Serum dilutions start at 1:20, visualized by the dotted line. Results are grouped by variant **(A-C)** or by infection/vaccination status **(D-F)** for clarity. Statistical analysis was performed using Kruskal-Wallis Multiple Comparisons. I, Infection; V, Vaccine; IC50, half maximal inhibitory concentration. *p ≤ 0.05, **p ≤ 0.01, ***p ≤ 0.001, ****p ≤ 0.0001.

When comparing viral neutralization across variants, at visit 1, the median IC50 was highest for WT and significantly lower for both Delta and Omicron (**Figure 3A**). At visits 2 and 3, neutralization was significantly lower for Omicron compared to both WT and Delta for all groups (**Figures 3B, C**). WT neutralization was also significantly better than Delta neutralization in most subjects at all visits (**Figures 3A-C**). For Omicron, median IC50 was >100 only in the I+/V+ groups at visits 2 and 3 (**Figures 3D-F**).

### Neutralization was more durable in the setting of prior infection compared to only vaccination

**Figure 4:**
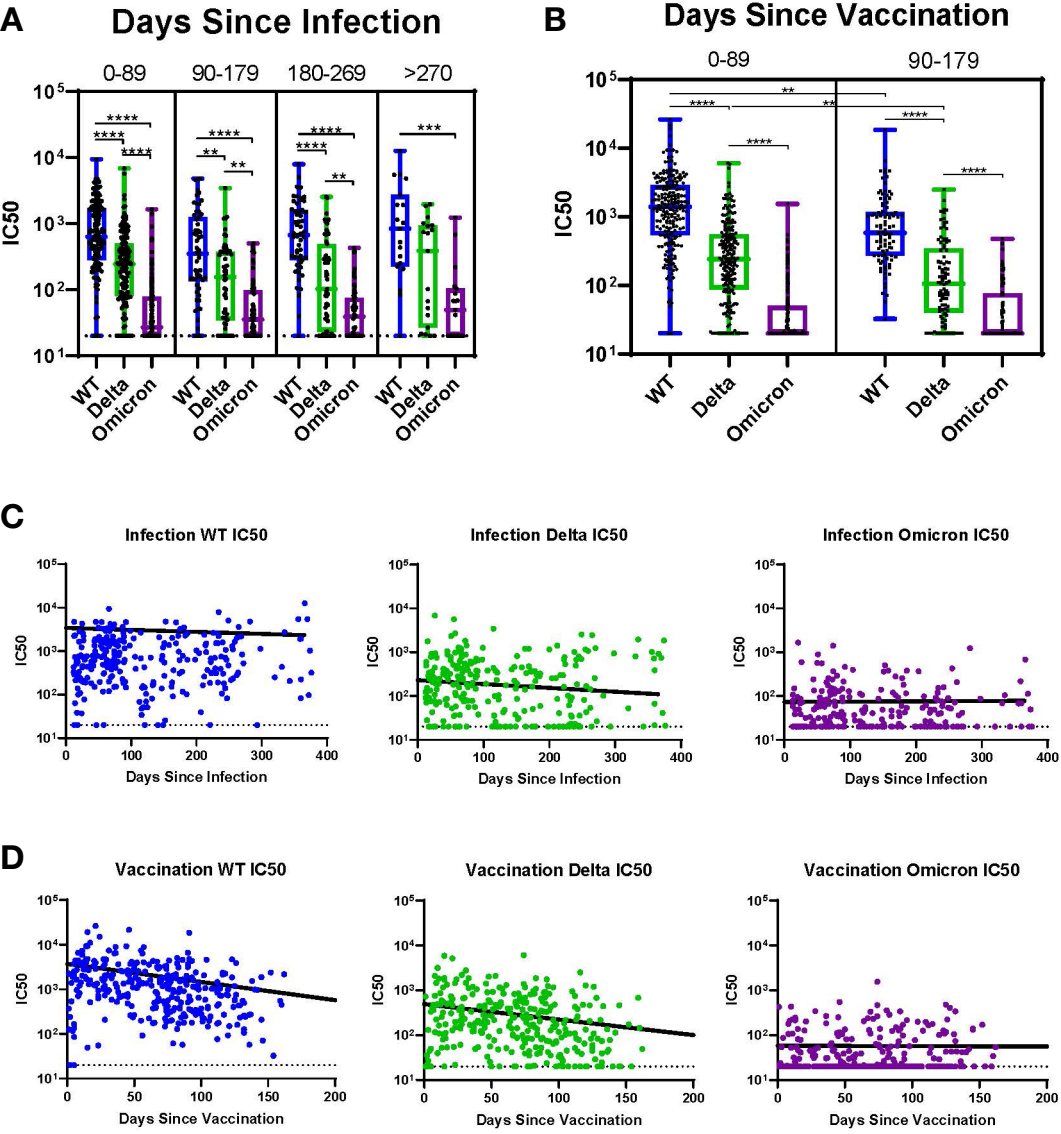
Impact of Time Since Infection or Vaccination. **(A)** Individuals at all timepoints with history of infection but without vaccination analyzed over time across all variants. **(B)** Individuals at all timepoints with history of vaccination but without prior infection analyzed over time across all variants. **(C)** Individual IC50s over time by variant for all participants with history of infection without vaccination. Line indicates the results of a linear mixed effects model of log-transformed IC50 values analyzing the role of time since infection controlling for vaccine status. **(D)** Individual IC50s over time by variant for all participants with history of vaccination without prior infection. Line indicates the results of a linear mixed effects model of log-transformed IC50 values analyzing the role of time since vaccination controlling for infection status. **p ≤ 0.01, ***p ≤ 0.001, ****p ≤ 0.0001.

Subjects with history of infection without vaccination (I+/V-) were analyzed over time across all variants and sorted into 90-day intervals since the time of infection (**Figure 4A**). In these individuals there were no significant differences in the median IC50 across all 90-day intervals for all variants (WT, Delta, and Omicron); this extended out to >270 days. For all intervals except >270 days, the median IC50 value for WT was significantly greater than for Delta. In contrast, at all timepoints the median IC50 for both WT and Delta were significantly higher than Omicron. Among individuals with a history of vaccination without infection (I-/V+), the time since vaccination had a much greater impact on neutralization titers (**Figure 4B**). In these subjects there was a significant decline in median IC50 for both WT and Delta even after 90 days. Neutralization titers against Omicron in these subjects were initially low so no trend could be determined. The median IC50 for WT was significantly greater than Delta for both time intervals.

We utilized linear mixed effects models of log-transformed IC50 values across time for each variant to further evaluate the trend of IC50 values since either infection or vaccination. **Figure 4C** shows a linear mixed effects model of log-transformed IC50 values superimposed on the IC50 values for each variant analyzing the role of time since infection, controlling for vaccine status. There was a slight decline in IC50 over time for both WT and Delta, but this was not observed for Omicron. **Figure 4D** shows a linear mixed effects model of log-transformed IC50 values analyzing the role of time since vaccination, controlling for infection status. There was a sharp decline in IC50 over time for both WT and Delta, but no neutralization was observed for Omicron.

### Correlation between clinical testing and neutralization results

**Figure 5:**
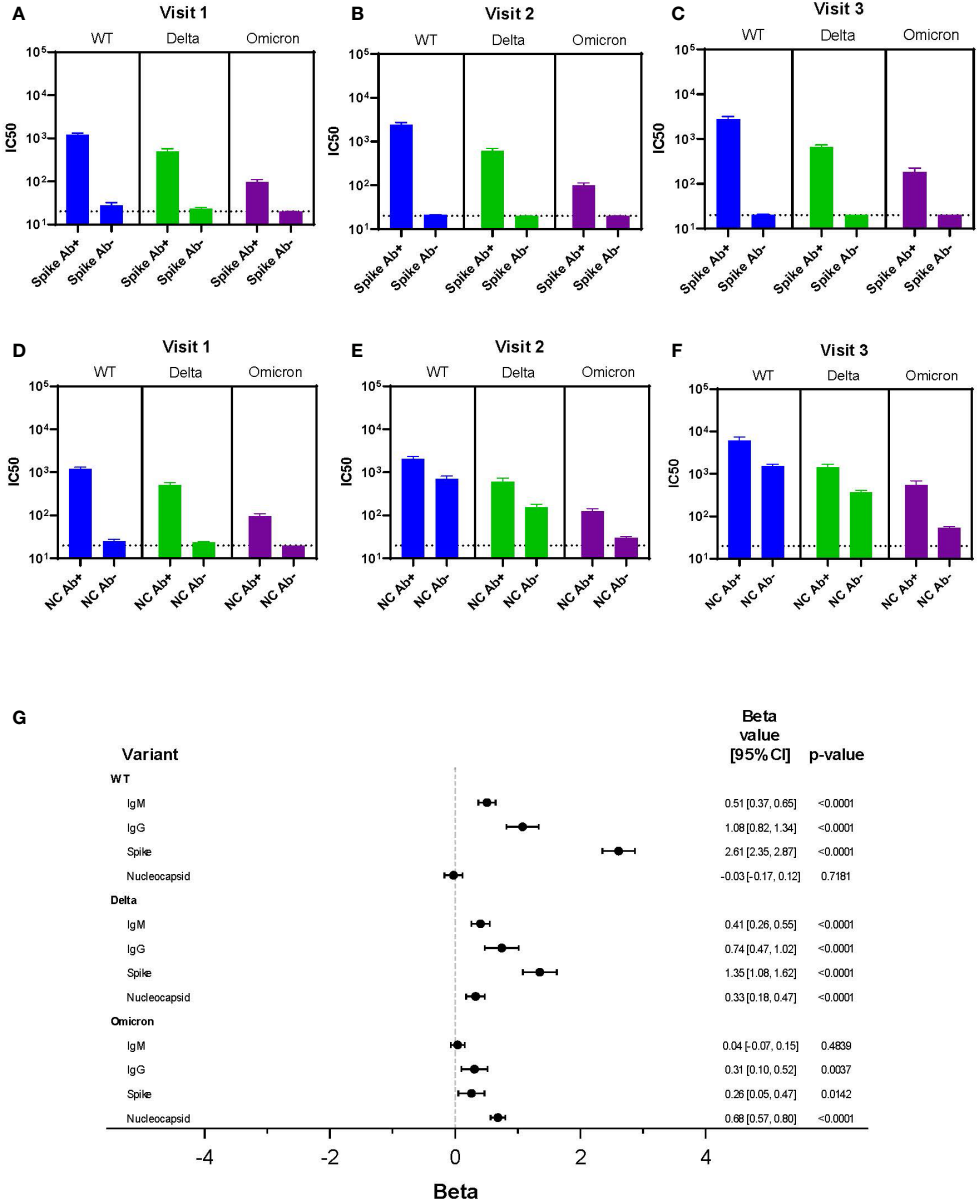
Correlation Between Clinical Testing and Viral Neutralization. **(A-F)** IC50 results for each variant (WT, Delta, Omicron) categorized by spike antibody and nucleocapsid status (positive or negative) regardless of infection or vaccination status across all variants and time points. Serum dilutions start at 1:20, visualized by the dotted line. **(G)** Results of a linear mixed effects model of log-transformed IC50 values analyzing LFA results each for IgM and IgG, spike antibody status, and nucleocapsid antibody status all as dichotomous results.

We sought to define whether readily available clinical serological assays predicted neutralization results. Spike antibody levels were associated with IC50 results across variants at each visit, but the poorest association was for Omicron (**Figures 5A-C**). Nucleocapsid antibodies did not correlate with viral neutralization as well as spike antibodies in part because nucleocapsid antibodies were not present in individuals who were vaccinated and not infected. Therefore, at visit 2 and 3, the IC50 was similar for WT and Delta regardless of positive nucleocapsid testing, but the mean IC50 was higher for nucleocapsid positive individuals with Omicron (**Figures 5D-F**).

A multivariable linear mixed effects model of log-transformed IC50 values was used to assess each independent marker of clinical testing on every variant’s IC50 (**Figure 5G**). Clinical testing included spike and nucleocapsid antibodies, as well as presence of IgG and IgM using the LFA. For WT, the IgM, IgG, and spike antibody results were all independently associated with neutralization activity; the strongest association being with spike antibody (beta = 2.61). Of interest, there was no association seen between neutralization activity and nucleocapsid antibody resulting from infection in the WT data. A strong association between spike antibody and neutralization activity was also observed with the Delta variant (beta = 1.35). However, unlike WT virus nucleocapsid antibody was also significantly associated with neutralization, albeit to a lesser extent. This suggested augmentation of neutralization activity from infection.

In contrast, with the Omicron variant, nucleocapsid antibody was the most significant predictor of neutralization (beta = 0.68). This suggested infection was more important than vaccination for neutralizing activity. However, the association of nucleocapsid antibody with Omicron neutralization was lower than the associations between spike protein with WT and Delta neutralization activity. IgG (LFA) and spike antibody positivity were independently associated with Omicron variant neutralization, but these associations were minimal and unlikely to have clinical relevance. Together, this suggested vaccine immunity alone was associated with little neutralization activity against Omicron.

## Discussion

We studied viral neutralization of SARS-CoV-2 during the initial period of the pandemic, in which the only identified circulating virus was WT, to understand how this might relate to immunity against subsequent variants and protection against infection (19). There were several interesting findings that may have had impact on the progress of the pandemic. Viral neutralization activity was strong after either vaccination or infection and appeared to be boosted in those with a combination of both immune challenges. This correlated with the highly effective protection against clinical infection observed with vaccination during the WT and subsequent Delta variant infection cycles (4, 5). It also suggests why Delta infections mainly involved unvaccinated individuals with little evidence of reinfection during that period (22). In contrast, the Omicron wave of infection were noted for breakthrough infections in vaccinated individuals and reinfections (23, 24). While viral spike protein changes in Omicron may have been responsible for these events, our data demonstrate that in the absence of prior infection waning neutralizing immunity from vaccination also may have played a role. Thus, initial immune profiles in the pandemic provide insights into what drove subsequent waves of infection.

Viral neutralization activity was highest across all variants for those who were both infected and vaccinated. This provides evidence supporting reports of enhanced immunity from this combination against WT virus and the Delta variant (25, 26). While neutralization activity was significantly lower against Omicron, the infected and vaccinated group had a median IC50 over 100. This argues against the concern that initial COVID exposure limits subsequent immune response to variant SARS-CoV-2 viruses (27). Also, neutralization activity in the post-infection WT group may be better with Omicron than what we demonstrated since infection yields immunity to multiple viral proteins while pseudoviral neutralization measures neutralization specifically with spike protein. This is reinforced by the correlation between nucleocapsid antibody titers and neutralization activity for Omicron. On the other hand, a recent study suggests that Omicron infection in the absence of vaccination is ineffective for cross-variant immunity, supporting the need for vaccination even after infection (28). Our results clearly indicate that neutralization is more durable in the setting of infection compared to 2-dose vaccination alone, consistent with smaller prior studies of mRNA vaccines (23). Together, these findings support the use of Omicron vaccine boosters regardless of infection status.

This work also demonstrates conclusively that neutralization activity falls progressively from either infection or vaccination regardless of SARS-CoV-2 variant. Prior studies have shown somewhat similar findings in smaller cohorts (13, 29, 30), and the present work is notable for its large cohort size and the fact that it utilized only WT infected or vaccinated individuals. These results also help explain the phenomenon of ‘breakthrough’ infections with Omicron, both after vaccination as well as previous infection (23, 24). Since much of the population was exposed to WT virus and/or vaccine (31), the results of this study are relevant for a substantial portion of the global population and, given the ubiquity of Omicron exposure (32, 33), re-creating the immune status of our cohort will be impossible.

Given the difficulties in performing SARS-CoV-2 neutralization or cellular immunity tests, identifying correlates of protection from available humoral testing is important (34). Since all vaccines against SARS-CoV-2 immunize with spike protein (4, 5), spike antibody levels after vaccination are elevated and may not reflect neutralizing antibody activity (35). Prior work also suggests that total SARS-CoV-2 antibody levels may predict risk for breakthrough infections (36). In this study, we were able to associate both infection and immune status to neutralization capacity, providing a stratification approach from clinical antibody testing for risk of infection. Also, given the many individuals infected during recent Omicron waves, nucleocapsid responses may be important for understanding future COVID-19 infection risk.

Despite our large and diverse cohort, our study has limitations. All participants that were both infected and vaccinated were always infected prior to vaccination. We cannot determine whether individuals vaccinated and subsequently infected would have the same neutralization profiles. Furthermore, booster doses were not available during the study period. However, most individuals in the United States are vaccinated adults without booster shots, so our data remain relevant.

In conclusion, individuals with a history of infection followed by vaccination have higher neutralizing antibody levels for all variants compared to those with vaccination or infection alone. Among individuals infected or vaccinated with WT SARS-CoV-2, viral neutralization capacity was highest for WT and Delta and was much lower for Omicron. Importantly, SARS-CoV-2 vaccine induced immunity wanes significantly over time but when combined with infection is much more persistent. Infection status and clinical antibody testing can provide insight into SARS-CoV-2 neutralization capacity, which is useful given the logistic constraints of neutralization testing. These findings help to explain the high rates of vaccine breakthrough infection observed with the Omicron variant and support the use of vaccine boosters after infection.

## Data availability statement

The datasets presented in this study can be found in online repositories. The names of the repository/repositories and accession number(s) can be found in the article/Supplementary Material.

## Ethics statement

The studies involving human participants were reviewed and approved by Human Research Protection Program/Institutional Review Boards- Office of Research Compliance Review, University of Michigan. Written informed consent to participate in this study was provided by the participants’ legal guardian/next of kin.

## Author contributions

KO’S, CS, JC, JT, PW, DO’S, KC, WP, CG, DM, RV, JLB, and JRB made substantial contributions to conception and design, acquisition of data, or analysis and interpretation of data. KO’S and CS drafted the article and JC, JT, PW, RV, JLB, and JRB reviewed it critically for important intellectual content. KO’S, CS, JC, JT, PW, DO’S, KC, WP, CG, DM, RV, JLB, and JRB gave final approval of the version to be published; and KO’S, CS, JC, JT, PW, DO’S, KC, WP, CG, DM, RV, JLB, and JRB agree to be accountable for all aspects of the work related to its accuracy or integrity. All authors contributed to the article and approved the submitted version.
